# A Pilot Study of Preoperative Submandibular POCUS to Predict Difficult Mask Ventilation

**DOI:** 10.24908/pocusj.v11i01.19821

**Published:** 2026-04-22

**Authors:** Jevaughn Davis, Erica Chemtob, Dora Lin, Esther Lee, Marianne David, Ryan Keneally, Anita Vincent, Eric Heinz

**Affiliations:** 1 Attending Physician, Assistant Professor, Department of Anesthesiology and Critical Care, George Washington School of Medicine and Health Sciences, Washington DC, USA; 2 Resident Physician, Department of Anesthesiology and Critical Care Medicine, The George Washington School of Medicine and Health Sciences, Washington DC, USA; 3 Medical Student, The George Washington School of Medicine and Health Sciences, Washington DC, USA; 4 Resident Physician, Department of Otolaryngology, George Washington School of Medicine and Health Sciences, Washington DC, USA; 5 Professor, Department of Anesthesiology and Critical Care, George Washington School of Medicine and Health Sciences, Washington DC, USA; 6 Attending Physician, Associate Professor, Department of Anesthesiology and Critical Care, George Washington School of Medicine and Health Sciences, Washington DC, USA

**Keywords:** Difficult mask ventilation, STOP-BANG, POCUS, Airway POCUS, Hyomental distance, Obstructive sleep apnea, Submandibular ultrasound

## Abstract

**Background:**

Multiple assessment tools are available to evaluate for difficult mask ventilation. Obstructive sleep apnea (OSA) is a known risk factor. In this observational study, patients were screened using the STOP-BANG questionnaire. Submandibular point of care ultrasound (POCUS) was performed to determine the correlation between ultrasound findings and difficult mask ventilation.

**Methods:**

POCUS was performed to measure tongue thickness (TT), geniohyoid muscle thickness (GMT), distance between lingual arteries (DLA), lateral pharyngeal wall thickness (LPW), hyomental distance (HMD), and oral cavity height (OCH). The results were analyzed to determine the correlation with difficult mask ventilation scores.

**Results:**

TT, GMT, DLA, and HMD were larger in those with STOP-BANG scores ≥ 3. OCH was larger for those with STOP-BANG score ≥ 5. TT was greater when mask ventilation required oral airway adjunct (p = 0.044) or management by two anesthesia practitioners (p = 0.006). The DLA was higher when mask ventilation required management by two anesthesia practitioners (p < 0.001) and higher when an oral airway adjunct was required (p = 0.056). Increased TT was associated with a higher mask ventilation score (odds ratio [OR] 1.152, 95% confidence interval [95%CI] 1.011–1.314, p = 0.034). A larger DLA was associated with increased odds of difficult mask ventilation (OR 1.207, 95% CI 1.1–1.324, p < 0.001).

**Conclusions:**

Submandibular POCUS could potentially be used to identify difficult mask ventilation in patients suspected of having OSA based on the STOP-BANG questionnaire.

## Introduction

Mask ventilation of an apneic patient is an essential component of airway management. The American Society of Anesthesiologists (ASA) considers mask ventilation difficult when an anesthesiologist cannot maintain an oxygen saturation of greater than 90% using 100% oxygen during positive pressure ventilation without assistance [1]. Assessment tools such as the Mallampati classification, mouth opening, and upper lip bite are widely used to evaluate the airway to predict difficulty in airway management. These tools have variable predictive value, and unanticipated difficult mask ventilation may still occur despite thorough use during preoperative clinical evaluation.

Certain patient conditions, such as obstructive sleep apnea (OSA), may increase the risk for difficult mask ventilation [2,3]. Patients with OSA are three to four times more likely to experience difficult mask ventilation, intubation, or both compared to patients without OSA [4]. Most cases of OSA are undiagnosed and untreated despite a high prevalence in the population [5]. The STOP-BANG questionnaire is a validated screening tool used to identify individuals at high risk for OSA. The presence of snoring, daytime somnolence, observed apnea, hypertension, body mass index (BMI) greater than 35 kg/m^2^, age over 50 years, neck circumference greater than 40 cm, and male gender have been positively correlated with an OSA diagnosis [6]. Scores range from 0 to 8 and scores of 0 to 2 are considered low risk, scores 3 to 4 moderate risk, and scores of 5 or higher are considered high risk for moderate to severe OSA [7]. The STOP BANG questionnaire has been validated for identifying patients at risk for OSA in the perioperative period as well [8].

Point of care ultrasound (POCUS) is increasingly used perioperatively for airway assessment but the techniques are novel and studies are limited [8,9]. Previous studies have suggested that submandibular POCUS measurements of the oral cavity can help predict difficult mask ventilation, particularly tongue thickness (TT) and the distance between lingual arteries (DLA) [10,11]. Other studies have found a correlation between TT, geniohyoid muscle thickness (GMT), lateral pharyngeal wall thickness (LPW), and the DLA with the severity of OSA [11,12].

To test these observations, patients were screened for OSA using the STOP-BANG questionnaire and those at high risk were identified based on their STOP-BANG score. Anatomical airway measurements were then assessed using POCUS. We hypothesized that patients with increased submandibular POCUS measurements are correlated with a higher risk for OSA and, consequently, for difficult mask ventilation. The goal of this study was to determine if point of care airway ultrasonography is a reliable diagnostic tool for identifying patients who will have difficult mask ventilation.

## Methods

This study was approved by the George Washington Institutional Review Board as a prospective observational study. Patients aged 18 years or older undergoing planned elective surgery with planned utilization of general anesthesia requiring positive pressure ventilation by mask were included. Exclusion criteria included patients with relative contraindications to mask ventilation, such as known recent food ingestion, pregnancy, severe gastroesophageal reflux, severe hiatal hernia, or those undergoing an emergency procedure. Recorded demographic information included age, sex, BMI, neck circumference, history of OSA, continuous positive airway pressure (CPAP) use, Mallampati classification, STOP-BANG score, and comorbid conditions.

Ultrasonographic images were obtained using a 3 to 8 MHz curvilinear probe and a SonoSite X-porte ultrasound system (FujiFilm, Philips Healthcare, Bothell, WA). Patients were scanned preoperatively in the supine position and had six airway dimensions evaluated. The six measurements taken were TT, GMT, DLA, LPW, hyomental distance (HMD), and oral cavity height (OCH). Measurements were made in real time while examining the patients, and the images were saved.

Patients were preoxygenated and underwent intravenous (IV) induction. Agents used for induction were not standardized and were determined by the anesthesia provider. Mask ventilation was initiated by the anesthesia provider, and the level of difficulty was evaluated. Mask ventilation difficulty level was determined using the scoring system proposed by Han et al [2]. A score of 1 was assigned for successful mask ventilation without assistance, 2 for mask ventilation requiring an airway adjunct, 3 for mask ventilation requiring two providers, and 4 for unable to mask ventilate [13]. Successful mask ventilation was defined as the sustained presence of end tidal carbon dioxide (ETCO_2_) and capnography waveform.

De-identified data were recorded using Microsoft Excel (Microsoft Corp, Redmond, WA). Descriptive analyses, including frequency and measures of central tendency such as mean and standard deviations, were performed using Statistical Product and Service Solutions (International Business Machines, Armonk, NY), version 28. Chi-square and Pearson's correlation were used to determine the association between categorical patient characteristics (e.g., sex, BMI by category, and STOP-BANG score by cut score) and mask ventilation score. The Kruskal-Wallis test was used to examine the relationship between airway POCUS measurements and STOP-BANG scores as seen in Tables 1 and 2. Multivariable logistic regression was used to determine the association between independent variables and ventilation score. A p-value of less than 0.05 was considered significant.

**Table 1.** Submandibular point of care ultrasound (POCUS) measurements by STOP-BANG score.

Anatomy	N	STOP-BANG score			p-value
		0–2	3–4	≥5	
TT	90	4.64 (0.39)	4.96 (0.45)	5.40 (0.52)	< 0.001
GMT	87	1.58 (0.28)	1.79 (0.35)	1.76 (0.48)	0.014
DLA	90	2.77 (0.63)	3.22 (0.72)	3.35 (0.68)	< 0.001
LPW	90	2.41 (0.61)	2.67 (0.74)	2.68 (0.83)	0.287
HMD	88	4.61 (0.57)	4.95 (0.58)	5.06 (0.57)	0.009
OCH	50	5.59 (0.56)	5.94 (0.49)	6.48 (0.51)	0.002

Values are mean (SD) in centimeters. p-values determined using the Kruskal-Wallis test among STOP-BANG score categories. DLA: distance between lingual arteries; GMT: geniohyoid muscle thickness; HMD: hyomental distance; LPW: lateral pharyngeal wall; OCH: oral cavity height; SD: standard deviation; TT, tongue thickness.

**Table 2.** Kruskal-Wallis pairwise comparisons between STOP-BANG categories.

STOP-BANG categories	TT	GMT	DLA	HMD	OCH
0–2 and 3–4	0.008	0.044	0.003	0.018	0.125
0–2 and >5	< 0.001	0.008	0.001	0.009	< 0.001
3–4 and >5	0.014	0.804	0.55	0.536	0.050

Values shown are p-values. Significant difference at < 0.05 adjusted by the Bonferroni correction for multiple tests. DLA: distance between lingual arteries; GMT: geniohyoid muscle thickness; HMD: hyomental distance; LPW: lateral pharyngeal wall; OCH: oral cavity height; SD: standard deviation; TT, tongue thickness.

## Results

There were 92 patients enrolled in the study, and 91 patients were included in the final analysis. One patient was excluded after consenting to participate, but preoperative POCUS was not performed. The mean age of enrolled patients was 51.3 years, standard deviation (SD) 17.3 years (Table 3). Of those enrolled, 62% were female and 18% had a history of OSA. Nearly half (48%) were obese (BMI ≥ 30), 26% were overweight (BMI 25–30), and 25% had a BMI between 18.5 and 25. There was a 50% prevalence of a STOP-BANG score of 3 or greater. In the cohort, 66% of patients had a mask ventilation difficulty score of 1, 28% had a score of 2, and 7% had a score of 3. No patients had a score of 4.

**Table 3.** Patient Characteristics. (n = 91).

Characteristics	n (%)
**Age, years (n = 81)**	
Mean ± SD	51.3 (17.3)
Range	19–89
**Sex (n = 81)**	
Male	31 (38.3)
Female	50 (61.7)
**BMI, kg/m^2^ (n = 91)**	
Mean ± SD	30.9 (7.9)
Range	18.8–56.8
18.5–24.9	23 (25.3)
25–29.9	24 (26.4)
30–34.9	14 (15.4)
>35	30 (33.0)
**Neck circumference, cm (n = 89)**	
Mean ± SD	39.7 (5.41)
Range	28.0–55.9
**History of OSA (n = 91)**	16 (17.6)
**Modified Mallampati score (n = 90)**	
1	32 (35.6)
2	28 (31.1)
3	21 (23.3)
4	9 (10.0)
**STOP-BANG score (n = 90)**	
0–2: low OSA risk	45 (50.0)
3–4: moderate OSA risk	28 (31.1)
≥5: high OSA risk	17 (18.9)
**Mask ventilation score (n = 91)**	
1: easy to MV	60 (65.9)
2: requiring oral airway	25 (27.5)
3: requiring 2 practitioners	6 (6.6)
4: unable to MV	0 (0)

BMI: body mass index; MV: mask ventilate; OSA: obstructive sleep apnea.

Age and sex were not significantly associated with difficult mask ventilation (p = 0.2 and p = 0.5, respectively). A STOP-BANG score greater than 3, representing higher risk of OSA, was associated with a higher mask ventilation score (p < 0.01).

Patients with STOP-BANG scores indicating moderate or higher risk of OSA (≥ 3) had significantly increased TT, GMT, DLA, and HMD (Table 4 and 5). Those with a STOP-BANG score ≥ 5 had significantly increased OCH, compared to those with STOP-BANG scores between 0 and 4.

**Table 4.** Submandibular point of care ultrasound (POCUS) measurements by mask ventilation score.

Anatomy	N	Mask ventilation score			p-value
		1	2	3	
TT	91	4.77 (0.48)	5.01 (0.49)	5.43 (0.60)	0.007
GMT	88	1.64 (0.33)	1.83 (0.39)	1.78 (0.48)	0.135
DLA	91	2.79 (0.59)	3.50 (0.70)	3.40 (0.79)	0.001
LPW	91	2.36 (0.63)	2.91 (0.71)	2.95 (0.77)	0.003
HMD	89	4.74 (0.59)	4.92 (0.56)	4.83 (0.81)	0.523
OCH	50	5.75 (0.61)	6.18 (0.59)	6.32 (0.30)	0.069

Values are mean (SD) in centimeters. p-values were determined using the Kruskal-Wallis test among mask ventilation scores. DLA: distance between lingual arteries; GMT: geniohyoid muscle thickness; HMD: hyomental distance; LPW: lateral pharyngeal wall; OCH: oral cavity height; SD: standard deviation; TT, tongue thickness.

**Table 5.** Kruskal-Wallis pairwise comparisons between mask ventilation scores.

Mask ventilation score pair	TT	DLA	LPW
1–2	0.044	0.056	0.002
1–3	0.006	< 0.001	0.067
2–3	0.128	0.712	0.916

Values shown are p-values. Significant difference at < 0.05 adjusted by the Bonferroni correction for multiple tests. DLA: distance between lingual arteries; GMT: geniohyoid muscle thickness; HMD: hyomental distance; LPW: lateral pharyngeal wall; OCH: oral cavity height; SD: standard deviation; TT, tongue thickness.

Patients with a mask ventilation score of 2 or 3 had significantly greater TT and LPW (Table 6). Patients with mask ventilation scores of 2 or greater also had significantly increased DLA compared to those with a score of 1.

**Table 6.** Multivariable logistic regression of key characteristics and mask ventilation scores.

	B	Odds ratio [95%CI]	p-value
BMI	0.018	1.018 [1.01–1.027]	<0.001
TT	0.142	1.152 [1.011–1.314]	0.034
DLA	0.188	1.207 [1.1–1.324]	<0.001

95%CI: 95% confidence interval; BMI: body mass index; DLA: distance between lingual arteries; TT, tongue thickness.

Increased BMI, TT, and DLA were associated with significantly increased probability of a higher mask ventilation score in a multivariate logistic regression model (Table 6). A BMI score greater than 30 was significantly associated with a mask ventilation score of ^3^2 (odds ratio [OR] 1.02, 95% confidence interval [95%CI] 1.01–1.03, p < 0.01). TT greater than 0.134 was associated with higher odds of mask ventilation score 2 (OR 1.15, 95%CI 1.01–1.31, p = 0.03). DLA greater than 0.188 was also associated with mask ventilation score 2 (OR 1.21, 95%CI 1.1–1.32, p < 0.01).

## Discussion

Difficulty in airway management can occur when there is an inability to mask ventilate, inability to intubate, or both. Inadequate mask ventilation may require an oral or nasal airway adjunct and/or a second practitioner to facilitate a two-handed technique. Our findings suggest POCUS may be useful in predicting difficult mask ventilation.

We demonstrated that preoperative submandibular POCUS can be used to measure several oral cavity parameters that correlate with increased difficulty in mask ventilation. Greater TT, DLA, and LPW measurements were observed among patients who experienced increased difficulty with mask ventilation. TT, DLA, and elevated BMI were independently associated with increased odds for difficult ventilation. Lin et al. have also studied ultrasound measurements and difficult mask ventilation, but found a correlation with higher tongue-base thickness and longer DLA, which were similar to our findings [11]. Similarly, Padhy et al. found that a DLA above 30.78 mm was the single most important predictor of difficult mask ventilation [14,15].

Beyond the current understanding of airway ultrasound evaluation and prediction of difficult mask ventilation, we found that GMT and HMD are increased in patients with mask ventilation scores of 2 compared to those with a score of 1. We did not find a difference when analyzing the entire group, including those with a mask ventilation score of 3. This could be due to a small sample size of four patients with a mask ventilation score of 3. A larger sample size would be needed to make a concrete statement on predictability of these variables for difficult ventilation. Further studies with larger sample sizes are needed for validity and generalizability to the general populace.

In addition to predicting difficulty with mask ventilation, it is important to evaluate for OSA in the perioperative period. While only TT and DLA were associated with increased risk for difficult mask ventilation, all airway POCUS measurements that were evaluated in this study correlated with a STOP-BANG score of 3 and increased risk for OSA. Prior studies showed that increased tongue base thickness was associated with increased OSA severity [16]. Our results suggest the clinical utility of airway POCUS may exceed just predicting difficulty with mask ventilation. POCUS evaluation of the airway in the preoperative period may help identify patients with OSA who are prone to airway collapse and obstruction throughout the perioperative period.

Airway POCUS can be time consuming and requires expertise compared to utilizing the STOP-BANG questionnaire to screen patients. STOP-BANG has been validated to predict difficult mask ventilation [9]. Similarly, in our study, we found a correlation between STOP-BANG and mask ventilation scores (p = 0.002). Khan et al. found that the STOP-BANG score has a high negative predictive value, which can be used to rule out difficult mask ventilation. In their study, 39.5% of patients with a STOP-BANG score of 3 or more were difficult to mask ventilate, compared to 7.5% in patients with a score less than 3 [16]. While a STOP-BANG evaluation is highly valuable and requires less skill and time, it is somewhat dependent on the reliability of patients self-reporting, which may be limited if patients are unaware of certain symptoms. Given the high incidence of OSA and the potential for patients to misreport symptoms, airway POCUS may have value. The additional time and expertise required for airway POCUS compared to utilizing the STOP-BANG questionnaire may yield a greater ability to screen for OSA.

Our study has some limitations. There is often anatomical variation between patients and obtaining an ideal view of the oral cavity (i.e., a clear outline of the tongue) may not always be possible. Moreover, experience levels of sonographers vary, which can affect the measurements that are obtained. In addition, we only looked at difficult mask ventilation which may not translate to difficult intubation. In fact, a recent study by Lin et al. suggested that submental ultrasound (i.e., using TT and DLA) was effective in predicting difficult mask ventilation but not difficult direct laryngoscopy [10]. Future studies will need to enroll a larger and more heterogeneous sample size to determine validity and applicability of our findings in the general population.

## Conclusion

Submandibular POCUS is a simple technique that can be used to assess upper airway anatomy. Higher scores on the STOP-BANG screening tool, an indicator of moderate-severe OSA, and higher measurements of certain airway parameters—specifically GMT and HMD—obtained on submandibular POCUS are correlated with difficult mask ventilation. Our report suggests there may be more parameters that can be used for identifying potential difficulty with mask ventilation. We also suggest airway POCUS may be valuable in screening for OSA in the perioperative period.
